# Exploration of Potential Target Genes of miR-24-3p in Chicken Myoblasts by Transcriptome Sequencing Analysis

**DOI:** 10.3390/genes14091764

**Published:** 2023-09-05

**Authors:** Xuanze Ling, Qifan Wang, Pengfei Wu, Kaizhi Zhou, Jin Zhang, Genxi Zhang

**Affiliations:** 1 College of Animal Science and Technology, Yangzhou University, Yangzhou 225009, China; lingxuanze@163.com (X.L.); 13851677739@163.com (Q.W.); wu_p_fei@163.com (P.W.); zkz282816@163.com (K.Z.); zjown0225@163.com (J.Z.); 2 Joint International Research Laboratory of Agriculture & Agri-Product Safety, Yangzhou University, Yangzhou 225009, China

**Keywords:** skeletal muscle, microRNA, DEGs, GO enrichment analysis, KEGG pathway analysis

## Abstract

Broiler skeletal muscle growth is significantly influenced by miRNAs. Our earlier research demonstrated that miR-24-3p significantly suppressed the proliferation of chicken myoblasts while promoting their differentiation. The purpose of this study is to investigate miR-24-3p potential target genes in chickens. We collected myoblasts of Jinghai yellow chicken and transfected four samples with mimics of miR-24-3p and another four samples with mimic NC (negative control) for RNA-seq. We obtained 54.34 Gb of raw data in total and 50.79 Gb of clean data remained after filtering. Moreover, 11,635 genes were found to be co-expressed in these two groups. The mimic vs. NC comparison group contained 189 DEGs in total, 119 of which were significantly up-regulated and 70 of which were significantly down-regulated. Important biological process (BP) terminology such as nuclear chromosomal segregation, reproduction, and nuclear division were discovered by GO enrichment analysis for DEGs in the mimic vs. NC comparison group. KEGG pathway analysis showed that focal adhesion, cytokine–cytokine receptor interaction, the TGF-β signaling pathway, and the MAPK signaling pathway were enriched in the top 20. Variation site analysis illustrated the SNP (single nucleotide polymorphisms) and INDEL (insertion–deletion) in the tested samples. By comparing the target genes predicted by miRDB (MicroRNA target prediction database) and TargetScan with the 189 DEGs found by the transcriptome sequencing, we discovered two up-regulated DEGs (*NEURL1* and *IQSEC3*) and two down-regulated DEGs (*REEP1* and *ST6GAL1*). Finally, we carried out qPCR experiments on eight DEGs and discovered that the qPCR results matched the sequencing outcomes. These findings will aid in identifying potential miR-24-3p target genes in chicken skeletal muscle and offer some new directions for upcoming research on broiler breeding.

## 1. Introduction

Chicken is a globally ubiquitous food that provides nutrition and health for humans [1]. With the improvement in people’s life quality, the consumption of chicken rises constantly and becomes a symbol of a healthy and low-carbon lifestyle. Up to now, slow growth rates and low feed utilization rates are still severe issues in cultivating local broiler breeds in China. Molecular breeding is utilized to hasten the selection of fast-growth traits to address these issues. It is well known that chicken myofiber develops in the early stages of embryogenesis and the total number of muscle fibers is almost fully established before hatching [2]. As a result, the development of the chicken’s skeletal muscle during the embryonic stage is crucial.

miRNAs are one kind of evolutionarily conserved non-coding RNA that takes part in the regulation of eukaryotic genes after transcription [3]. Lin-4 and Let-7, the first two identified miRNAs, were initially found to regulate the differentiation and division of stem cells in the nematode [4]. The translation of messenger RNAs (mRNAs) can be inhibited or their degradation promoted by miRNAs to modulate gene expression [5]. This finding suggests that miRNAs play a significant role in human diseases like cancer and cardiovascular and metabolic disorders. It has also been demonstrated that Drosophila melanogaster (D. melanogaster) contains various miRNAs that are associated with human disease [6]. In summary, miRNAs can be found to be conserved across various species.

The role of miRNAs in the regulation of numerous biological processes has recently come to light [7], including skeletal muscle development [8]. Various studies have revealed that miRNAs target specific genes and have an impact on biological function. According to Liu et al. [9], miR-200c-5p targeted *Adamts5* to influence myoblast migration and differentiation during the process of skeletal muscle regeneration. Wang et al. [10] identified that miR-34b-5p targeted *IGFBP2* to regulate the proliferation and differentiation of myoblasts. In our previous study, we collected the leg muscles from two groups of Jinghai yellow chickens (slow- and fast-growing groups) at the age of 300 days for miRNA sequencing to search for new miRNAs related to skeletal development. Through bioinformatics analysis, the outcome revealed that novel_miR_133 and miR-24-3p were two miRNAs that are significantly related to muscle development [11]. We also demonstrated that miR-24-3p inhibited myoblast proliferation while promoting their differentiation.

The discovery and study of miRNAs offer fresh insights into the research of skeletal muscle growth and development in chickens [12]. Jinghai yellow chicken is a well-known indigenous Chinese breed with good flavor and disease resistance [13]. The purpose of this study was to look for new miR-24-3p target genes and prepare for future research into its role in regulating the proliferation and differentiation of Jinghai yellow chicken myoblasts. In the experiment, we collected eight samples of Jinghai yellow chicken myoblasts and transfected four of them with miR-24-3p mimic and the other four with mimic NC. Finally, the total RNA was extracted to perform RNA-seq. We look forward to discovering some key genes related to the muscle development of chickens.

## 2. Materials and Methods

### 2.1. Ethics Statement

This study was fully consistent with the codes made by the Chinese Ministry of Agriculture. The animal experiments performed in the study were all evaluated and approved by the Animal Ethics Committee of Yangzhou University (202103298).

### 2.2. Isolation and Culture of Chicken Primary Myoblasts (CPMs)

As mentioned above, we used Jinghai yellow chickens as the laboratory animals in this experiment. Fertilized eggs were collected after artificial insemination and then hatched at 37 °C and 60% humidity. The leg muscle tissues were collected at embryonic stage 14 (E14) and the chicken primary myoblasts (CPMs) were extracted from the leg tissues. We removed the bones and blood from the leg muscle and then cut it into meat paste. We digested the meat paste with collagenase type I (Gibco, Grand Island, NY, USA) at 37 °C. After digestion, we filtered out the impurities in the mixture and cultured the CPMs in a 10 cm cell culture dish [11]. The culture medium we used was DMEM/F12 medium (Solarbio, Beijing, China) supplemented with 20% fetal bovine serum (Gibco, Grand Island) and 1% penicillin/streptomycin solution (Sangon Biotech, Shanghai, China) [11].

### 2.3. Transfections, RNA Extraction, Quantification, and Qualification

When the density of CPMs reached 60%, we transfected four samples with mimic and the other four samples with mimic NC, respectively. The reagents used for transfection were jetPRIME reagent (Polyplus, Illkirch, France) and 50 nM miR-24-3p mimic and mimic NC (GenePharma, Shanghai, China) [11]. Trizol (TIANGEN, Beijing, China) was added to these eight samples and the total RNA was extracted by standard methods [14]. Afterward, the Agilent 2100 bioanalyzer (Agilent, Santa Clara, CA, USA) was employed to assess the RNA integrity, with stringent quality control being applied to the RNA samples [15].

Sequences of the mimic (5′→3′), Sense: UGGCUCAGUUCAGCAGGAACAG. Antisense: GUUCCUGCUGAACUGAGCCAUU. Sequences of the mimic NC (5′→3′). Sense: UUCUCCGAACGUGUCACGUTT. Antisense: ACGUGACACGUUCGGAGAATT.

### 2.4. cDNA Library Preparation, Quality Inspection, and Sequencing

The RNA samples prepared for sequencing were the total RNA contained from 8 samples in the previous step. Enriched mRNA was obtained from the total RNA utilizing poly-T oligo-attached magnetic beads. Then, mRNA was randomly broken into fragments in fragmentation buffer. The fragmented mRNA was used as a template to produce the first strand of cDNA. Following that, the second strand cDNA synthesis was carried out using DNA Polymerase I and RNase H. We screened the cDNA fragments of 370~420 bp and purified them with the AMPure XP system (Beckman Coulter, Beverly, CA, USA) [16]. Then, PCR was performed and the PCR products were purified again by AMPure XP system to obtain the cDNA library. When the library construction was completed, the quantification was performed by Qubit2.0 Fluorometer. The concentration of the library was adjusted to 1.5 ng/µL. Subsequently, we applied the Agilent 2100 bioanalyzer to measure the insert size of the library. Finally, RNA-seq was performed on a NovaSeq 6000 platform (Illumina, San Diego, CA, USA).

### 2.5. Analysis of the Sequencing Results

Sequencing fragments were converted into raw reads from image data measured by the sequencer. The format of the raw reads was FASTQ. The sequence quality was checked (Q20, Q30, and GC content) and any low-quality reads were removed. The error rate was confirmed as low enough to enable successful downstream analyses. Bowtie 2 (v2.2.8) was used to align the paired-end clean reads to the GRCg6a reference genome [17]. Differential expression analysis between mimic and NC was carried out using DESeq2 [18]. Among all the genes tested in this study, those whose expression level showed *p*-adjust ≤ 0.05 were assigned as differentially expressed genes (DEGs). Moreover, the gene ontology (GO) enrichment and KEGG pathway analysis of the DEGs were implemented with GO-seq (R-3.3.2) and KOBAS (v2.0) [19,20], respectively. We determined the significant degree of GO enrichment and KEGG pathways by the standard of *p*-value ≤ 0.05 in the comparison group mimic vs. NC. Since variation site analysis was of great significance for learning about the protein function or gene-related diseases, we used GATK software (4.1.1.0) to perform SNP and INDEL variation site analysis on the RNA-seq data of these 8 samples; the SNP and INDEL variation sites were annotated by using SnpEff software (4.3) with default parameters [21]. The parameters of GATK are RMSMappingQuality (MQ) < 40.0, QualByDepth (QD) < 2.0, FisherStrand (FS) > 30.0, depth (DP) < 10, and quality (QUAL) < 20.

### 2.6. Real-Time Quantitative PCR of Differentially Expressed Genes

To help confirm the sequencing results, we conducted real-time fluorescence quantification experiments on 8 differentially expressed genes (DEGs). Primers of DEGs were designed with Primer 5.0 (Table S1). The company Tsingke (Nanjing, China) produced the primers of these 8 DEGs. The PCR products were detected by agarose gel electrophoresis. On the Applied Biosystems 7500 (ABI, Los Angeles, CA, USA), we performed RT-qPCR with three technical repetitions for each sample using the reagent Taq Pro Universal SYBR qPCR Master Mix (Q712, Vazyme Biotech Co., Ltd., Nanjing, China). We employed β-actin as the housekeeping gene in the RT-qPCR experiment and calculated the relative expression level of these 8 DEGs by means of the 2^−ΔΔCT^ method [22].

## 3. Results

### 3.1. Sequencing Data Quality Summary

We performed the RNA-seq experiment on eight samples of leg muscle from the Jinghai yellow chickens. The amount of raw data was 54.34 Gb and 50.79 Gb of clean data were obtained after quality control. The percentage of Q20 and Q30 reads was around 96% and 90%, respectively. The GC content in these eight samples was around 50% (Table 1).

**Figure 1:**
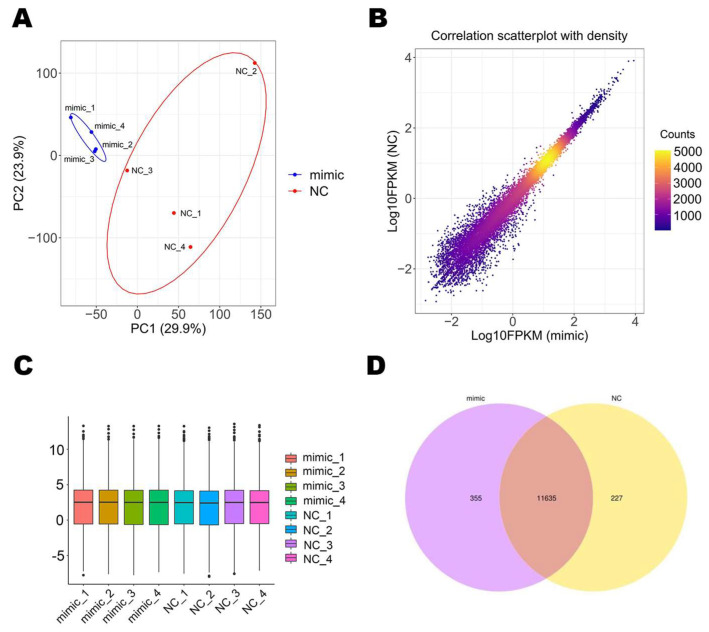
Sequencing data analysis. (**A**) The principal component analysis (PCA) of these 8 samples. (**B**) The correlation scatterplot with density. The colors from yellow to purple represent the intensity of FPKM values from high to low. (**C**) Boxplot of gene expression distribution of all examples. (**D**) Venn diagram of mimic vs. NC.

To assess the inter-group differences and sample duplication, we performed PCA analysis on the gene expression values of these eight samples and the result is shown in Figure 1A. In Figure 1B, the main scatter points are concentrated on the diagonal of the scatter plot, which demonstrated the repeatability and comparability of the sequencing results. Depending on the expression value (FPKM) of all genes, the condition of gene expression level distribution was shown through the box diagram (Figure 1C). The development of a Venn diagram showed that there were 355 genes uniquely expressed in the mimic group, 227 genes uniquely expressed in the NC group, and 11,635 genes that are co-expressed in these two groups (Figure 1D).

### 3.2. Statistics of Differentially Expressed Genes

**Figure 2:**
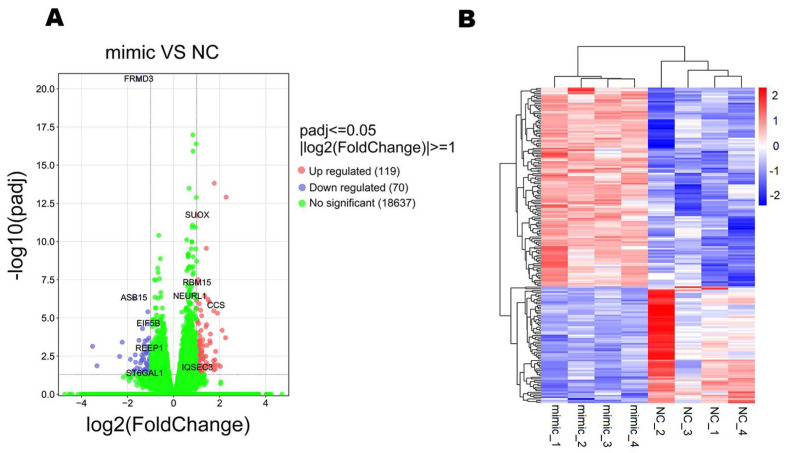
Statistics of DEGs in mimic vs. NC. (**A**) The Volcano plot of mimic vs. NC. (**B**) Clustering analysis of differentially expressed genes.

A total of 189 DEGs (Table S2) were identified with the standard of *p*-adjust ≤ 0.05 and |log2FoldChange| ≥ 1, including 119 up-regulated and 70 down-regulated genes (Figure 2A). The FPKM values of all DEGs were analyzed by the methods of hierarchical clustering and Z-Score normalization. The color in each square reflected the value obtained after homogenization in rows. According to the heatmap, we could clearly find that the DEGs and samples with similar expression patterns were grouped together (Figure 2B).

### 3.3. Functional Enrichment Analysis of the Differentially Expressed Genes

**Figure 3:**
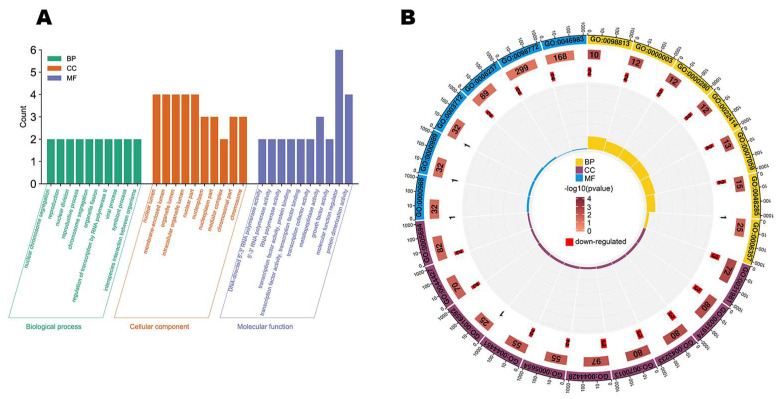
Gene ontology enrichment analysis. (**A**) The top 30 GO terms in mimic vs. NC. (**B**) The numbers of down-regulated genes and rich factors in GO enrichment terms.

GO function enrichment analysis found 32 significantly enriched GO terms (*p*-value ≤ 0.05) in mimic vs. NC. The biological process (BP) terms of the mimic vs. NC groups included nuclear chromosome segregation, reproduction, nuclear division, reproductive process, and so on (Figure 3A). Additionally, Figure 3A also included terms for molecular function (MF) and cellular component (CC). In addition, the numbers of down-regulated genes and rich factors in different GO enrichment terms are illustrated in Figure 3B.

**Figure 4:**
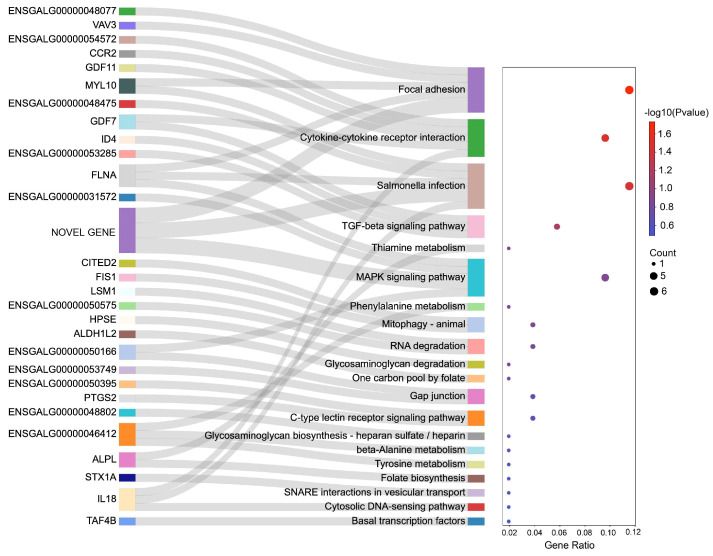
KEGG pathway analysis. The figure displays the top 20 pathways and their corresponding predicted target genes in the mimic vs. NC comparison group.

KEGG pathway analysis showed the top 20 KEGG pathways in the mimic vs. NC comparison groups, including focal adhesion, cytokine–cytokine receptor interaction, Salmonella infection, the TGF-β signaling pathway, thiamine metabolism, and the MAPK signaling pathway (Figure 4). Focal adhesion, cytokine–cytokine receptor interaction, and Salmonella infection pathways had *p*-values lower than 0.05. We could see the genes enriched in terms of pathways. For example, *VAV3*, *FLNA*, *ENSGALG00000048077*, and *MYL10* were enriched in terms of focal adhesion. *GDF11*, *GDF7*, *CCR2*, *IL18*, and *ENSGALG00000054572* were enriched in terms of the cytokine–cytokine receptor interaction. *ENSGALG00000048475*, *FLNA*, *MYL10*, and *IL18* were enriched in terms of Salmonella infection. *ENSGALG00000053285*, *ID4*, and *GDF7* were enriched in the TGF-β signaling pathway. Only *ALPL* was enriched in terms of thiamine metabolism. *ENSGALG00000031572*, *FLNA*, and *ENSGALG00000050166* were enriched in the MAPK signaling pathway.

### 3.4. Variation Site Analysis

The variation sites included SNP (single nucleotide polymorphisms) and INDEL (insertion–deletion). Generally speaking, SNP refers to single nucleotide variations with a variation frequency greater than 1%. INDEL refers to the insertion–deletion of small segments of the tested samples compared to the reference genome, which might contain one or more bases.

**Figure 5:**
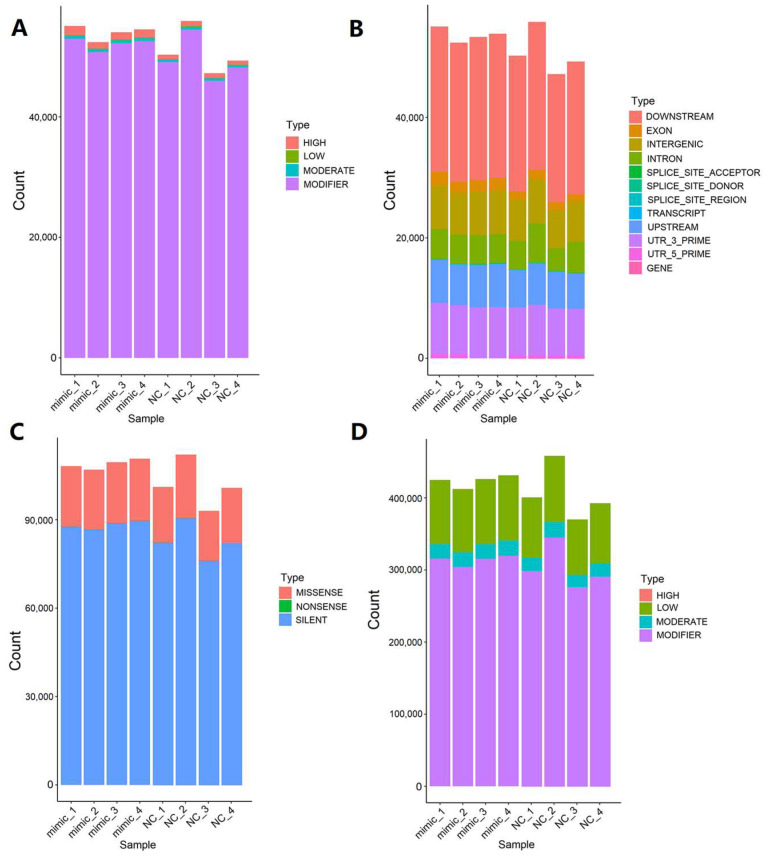
Variation site analysis. (**A**) INDEL (insertion–deletion) impact analysis. (**B**) INDEL region analysis. The downstream_gene_variant occurred in DOWNSTREAM. The frameshift_variant occurred in EXON. The intergenic_region occurred in INTERGENIC. The intron_variant occurred in INTRON. The splice_acceptor_variant occurred in SPLICE_SITE_ACCEPTOR. The splice_donor_variant occurred in SPLICE_SITE_DONOR. The splice_region_variant occurred in SPLICE_SITE_REGION. The non_coding_transcript_exon_variant occurred in TRANSCRIPT. The upstream_gene_variant occurred in UPSTREAM. The 3_prime_UTR_variant occurred in UTR_3_PRIME. The 5_prime_UTR_variant occurred in UTR_5_PRIME. Few variation sites were found in GENE. (**C**) SNP function analysis. (**D**) SNP impact analysis.

After using GATK (genome analysis ToolKit) to detect variation sites, we made statistics on each variation site according to SnpEff annotation information, mainly including variation site function statistics, variation site area statistics, and variation site impact statistics. The INDEL impact was analyzed from four aspects: HIGH, LOW, MODERATE, and MODIFIER (Figure 5A). It was obvious that the MODIFIER level was highest in these four levels. The INDEL annotation included 12 descriptors, as detailed in Figure 5B. It was clear that the downstream variants made up the largest proportion of the INDELs. The SNP function was counted and mapped from three aspects: missense variant, nonsense variant, and silent variant (Figure 5C). We could see that silent variants were most common, with a few nonsense variants highlighted. The impact of the SNPs was calculated and mapped using the four levels of HIGH, LOW, MODERATE, and MODIFIER (Figure 5D).

Finally, different types of INDEL variants like the downstream_gene_variant, 3_prime_UTR_variant, frameshift_variant, splice_region_variant, and intron_variant were found in *REEP1*. The impact of the frameshift_variant in *REEP1* was HIGH. The impacts of the splice_region_variant and intron_variant were LOW. The impacts of downstream_gene_variant and 3_prime_UTR_variant were MODIFIER. 3_prime_UTR_variant and intron_variant occurred in *NEURL1*. The impacts of 3_prime_UTR_variant and intron_variant were all MODIFIER. We also discovered 3_prime_UTR_variant and downstream_gene_variant in *ST6GAL1*. The impacts of 3_prime_UTR_variant and downstream_gene_variant were all MODIFIER. However, we did not find any kind of INDEL variant in *IQSEQ3*. More details are provided in Table S3.

For SNPs, the impact of downstream_gene_variant, 3_prime_UTR_variant, and intron_variant was MODIFIER in *REEP1*. The impact of the synonymous_variant was LOW in *REEP1*. The impact of the missense_variant was MODERATE in *REEP1*. The impact of 3_prime_UTR_variant, 5_prime_UTR_variant, upstream_gene_variant, downstream_gene_variant, and intron_variant was MODIFIER in *ST6GAL1*. While the impact of synonymous_variant in *ST6GAL1* was LOW, the impact of 3_prime_UTR_variant, intron_variant, and downstream_gene_variant was MODIFIER in *NEURL1*. While the impact of synonymous_variant was LOW in *NEURL1*, we did not find any kind of SNP variant in *IQSEQ3*. More details are provided in Table S4.

### 3.5. Target Gene Prediction and RT-qPCR Experiment Results

**Figure 6:**
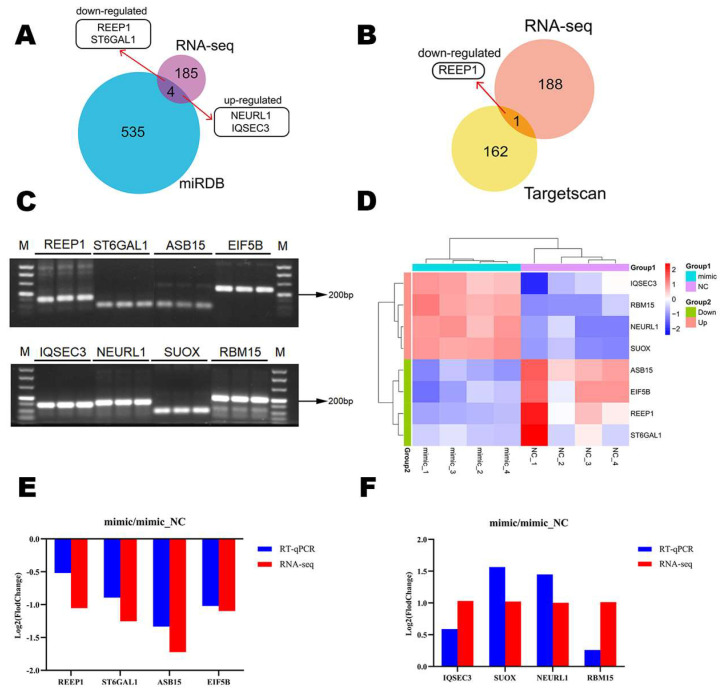
Target gene prediction and verification of the sequencing results. (**A**) Venn diagram showed the common genes between the target genes predicted by miRDB and the DEGs detected by sequencing. (**B**) The Venn diagram showed common genes between the target genes predicted by Targetscan and DEGs detected by sequencing. (**C**) Agarose gel results of 8 DEGs primers. Character “M” represented the marker. (**D**) Expression level of 8 DEGs in RNA-seq results. The colors from red to blue represent a level from high to low. (**E**) RT-qPCR and RNA-seq results of 4 down-regulated DEGs. (**F**) RT-qPCR and RNA-seq results of 4 up-regulated DEGs.

Four of these target genes predicted by miRDB were the same as the DEGs detected by RNA-seq, including two down-regulated genes *REEP1* and *ST6GAL1*, as well as two up-regulated genes *NEURL1* and *IQSEC3* (Figure 6A). The target genes predicted by Targetscan shared only *REEP1* with the DEGs detected by RNA-seq (Figure 6B). Based on the agarose gel electrophoresis results, the PCR product sizes of the primers for the 8 DEGs were consistent with those designed by Primer 5.0 (Figure 6C). According to the RNA-seq result, these 8 DEGs and samples were clustered into two groups (Figure 6D). In addition, we calculated the relative expression level of RNA-seq and that of RT-qPCR for the 8 DEGs in groups of mimic and NC with the standard of log2FoldChange (Figure 6E,F). The results showed that the tendency of changes in the 8 DEGs in the sequencing and quantitative results remains consistent, which proved that RNA-seq results were reliable. From these studies, we identified that *REEP1* and *ST6GAL1* may be the target genes of miR-24-3p.

## 4. Discussion

Chicken supplies essential proteins to humans all over the world and has the benefits of being high in protein, low in fat, and low in calories [23]. In addition, chicken can be a substitute for many other kinds of meat. Growth traits have always been the focus of attention in the development of livestock products. Skeletal muscle development is particularly crucial for progress in the broiler industry as it directly contributes to chicken production [24]. Studies of miRNAs offer fresh perspectives on the investigation of skeletal muscle development. Extensive experiments have been performed on miRNAs and their target genes to illustrate their functions in chicken skeletal muscle growth. Zhang et al. [25] observed that miR-10a-5p targeted the *BCL6* gene to regulate skeletal muscle growth, development, and autophagy. Zhang et al. [26] revealed that miR-21-5p targeted *KLF3* to regulate the proliferation and differentiation of skeletal muscle satellite cells in chicken. Cao et al. [27] found miR-99a-5p targeted at *MTMR3* to regulate the proliferation and differentiation of satellite cells in chicken.

In our earlier study, we discovered that miR-24-3p significantly inhibited the proliferation and promoted the differentiation of CPMs, which indicated that miR-24-3p correlated with muscle development. In this research, we overexpressed miR-24-3p and performed RNA-seq on CPM samples in order to look for potential target genes of miR-24-3p. According to the RNA-seq results, 119 up-regulated DEGs and 79 down-regulated DEGs were found in mimic vs. NC, including *REEP1*, *ST6GAL1*, and *ASB15*. We also predicted the possible target genes by miRDB and Targetscan. Using RNA-seq and target gene prediction, *REEP1* and *ST6GAL1* were the most likely genes targeted by miR-24-3p.

Moreover, we performed GO enrichment and KEGG pathway analysis to learn about the biological functions of these DEGs. We speculated that the target genes *REEP1* and *ST6GAL1* were involved in skeletal muscle development. For example, *REEP1* was reported to have the function of endoplasmic reticulum antistress [28]. In another study, Qin et al. [29] discovered that the up-regulation of *REEP1* can restore mitochondrial function to rescue motor neuron degeneration in SOD1G93A mice. Li et al. [30] found that *REEP1* could be transcriptionally activated by *p53*, which leads to an increase in Ca^2+^ in skeletal muscle mitochondria. In the Golgi compartment, *ST6GAL1* is a sialyl-transferase that mediates the glycosylation of proteins and lipids to generate functionally significant glycoproteins and glycolipids [31]. Extensive studies were performed to find that *ST6GAL1* was related to cancers [32,33]. However, few studies have been found about the relationship between *ST6GAL1* and skeletal muscle growth. It was reported that *ST6GAL1* was principally expressed in the liver, placenta, and skeletal muscle [34]. In our study, *ST6GAL1* was enriched in GO_BP entries like the glycoprotein biosynthetic process, macromolecule glycosylation, and glycoprotein metabolic process. These GO_BP entries were all related to ‘glycoprotein’. According to Osseni et al. [35], skeletal muscle fiber fragility and atrophy were caused by the disruption of the dystrophin-associated glycoprotein complex caused by the absence of dystrophin in Duchenne muscular dystrophy. This discovery illustrated that ‘glycoprotein’ had a relationship with skeletal muscle development. We therefore hypothesize that *ST6GAL1* is involved in skeletal muscle development. We also found that *ST6GAL1* was enriched in KEGG pathways such as N-glycan biosynthesis and other types of O-glycan biosynthesis. O-glycan was also shown to participate in the process of muscle development [36].

Apart from these two potential target genes, some other DEGs were also discovered to have relationships with skeletal muscle development. For instance, *ASB15*, which is primarily expressed in chicken skeletal muscle, is a member of the ankyrin repeat and suppressor of cytokine signaling box families [37]. Moreover, Wang et al. [38] discovered that the *ASB15* gene was expressed in different tissues of Gushi chickens such as the heart, breast muscle, and leg muscle. In another experiment, single-nucleotide variations (SNPs) in the *ASB15*’s flanking region were detected in Gushi chickens and the results suggested that *ASB15* had a remarkable impact on chicken performance traits [39]. In our study, *ASB15* was enriched in the intracellular signal transduction pathway. Intracellular signal transduction, for instance, the Notch signaling pathway, is a crucial regulator of skeletal muscle growth and regeneration [40]. Our result indicated that *ASB15* may function in skeletal muscle development. *NEURL1* was found to be related to reproduction traits in Bama Xiang pigs [41]. Li et al. [42] identified *NEURL1* as a biomarker of muscle growth in selective breeding programs for rice flower carp. As shown by reports, *RBM15* is vital to the development of cancer [43,44,45]. *RBM15* was discovered to be involved in the process of developing duck embryonic skeletal muscle in breast and leg muscle by Chen et al. [46] through qRT-PCR analysis.

Moreover, among all the KEGG pathways we found, the top six were focal adhesion, cytokine–cytokine receptor interaction, Salmonella infection, the TGF-β signaling pathway, thiamine metabolism, and the MAPK signaling pathway. Huang et al. [47] suggested that fat mass- and obesity-associated (*FTO*) genes promoted chicken myoblast differentiation via the focal adhesion pathway. Cui et al. [48] found that focal adhesion was closely associated with the growth of embryonic skeletal muscle in chickens. Metzger et al. [49] discovered that cytokine–cytokine receptor interaction was involved in myoblasts proliferating derived from piglets of different ages. We have found no previous studies on the relationship between Salmonella infection and skeletal muscle development. The TGF-β signaling pathway was proven to be a key regulator of skeletal muscle growth [50,51]. Studies provided evidence that the MAPK signaling pathway played a significant role in myoblast development. For example, Guan et al. [52] discovered that butyrate activated the ERK/MAPK pathway to promote C2C12 myoblast proliferation. Chen et al. [53] demonstrated that lncRNA Has2os regulated skeletal muscle differentiation by inhibiting the JNK/MAPK signaling pathway in mice. In our study, genes detected in these above-mentioned KEGG pathways have the potential to participate in regulating skeletal muscle proliferation and differentiation.

Through variation site analysis, we identified where mutations could be resulting in functional consequences in our identified candidate genes. *REEP1* and *ST6GAL1* are the two potential target genes of miR-24-3p. We identified an INDEL in *REEP1* which causes a frameshift mutation with predicted HIGH impact, which indicates that this variant may cause protein truncation or functional loss. No high-impact SNP variants were identified in *REEP1*. The impacts of INDEL and SNP variants discovered in *ST6GAL1* were MODIFIER, meaning that there was no evidence of the functional impact on *ST6GAL1*. A previous study showed that *REEP1* variants were related to hereditary spastic paraplegia (HSP) [54]. We predict that variants in *REEP1* may also be related to skeletal muscle development.

## 5. Conclusions

Through RNA-seq and bioinformatics analysis, we identified 189 DEGs in the miR-24-3p mimic vs. NC comparison group, 119 DEGs were significantly up-regulated and 70 others were significantly down-regulated. Based on the GO enrichment terms and KEGG pathways, we found some significant genes. BP terms of GO enrichment analysis found that *ENSGALG00000045898* and *ENSGALG00000002511* were included in nuclear chromosomal segregation, reproduction, and nuclear division in the mimic vs. NC comparison. group. Pathways including focal adhesion, cytokine–cytokine receptor interaction, and MAPK signaling were among the top 20 most enriched KEGG pathways in our analysis. In addition, the variation site analysis of the tested samples provided an important basis for genetic variation in chickens. Finally, we predicted that *REEP1* and *ST6GAL1* were potential target genes of miR-24-3p by combining sequencing results with software predictions as well as RT-qPCR validation. These findings will help to reveal how the predicted genes work with miR-24-3p in the development of chicken myoblasts.

## Figures and Tables

**Table 1:** Quality assessment of myoblast transcriptome sequencing data.

Sample	Raw Reads	Clean Reads	Error Rate	Q20	Q30	GC_ pct
mimic_1	46,980,614	44,438,956	0.03	96.08%	90.12%	51.65%
mimic_2	43,638,870	41,261,988	0.03	96.34%	90.68%	50.73%
mimic_3	46,386,290	44,033,654	0.03	96.41%	90.85%	51.03%
mimic_4	47,859,392	45,430,248	0.03	96.14%	90.30%	51.36%
NC_1	41,796,294	37,958,990	0.03	96.28%	90.42%	48.71%
NC_2	47,161,432	45,087,540	0.03	96.62%	91.18%	49.07%
NC_3	41,438,596	38,035,454	0.03	96.02%	89.94%	49.64%
NC_4	46,961,062	42,302,224	0.03	96.54%	90.92%	48.09%

## Data Availability

The raw data of the study have been uploaded to the sequence read archive (SRA) database; the accession number is PRJNA993809 (https://www.ncbi.nlm.nih.gov/bioproject/?term=PRJNA993809) (accessed on 12 July 2023).

## Supplementary Materials

The following supplemental information in this study can be downloaded at: https://www.mdpi.com/article/10.3390/genes14091764/s1, Table S1: Primers of 8 DEGs. Table S2: The list of 189 DEGs. Table S3: INDEL mimic NC. Table S4: SNP mimic NC.
