# Supplementary material for: Exploration of Potential Target Genes of miR-24-3p in Chicken Myoblasts by Transcriptome Sequencing Analysis
Source: Genes (Basel). 2023 Sep 5;14(9):1764. doi: 10.3390/genes14091764 (PMC10530709; doi:10.3390/genes14091764)
Supplement: Supplementary file 1 [file genes-14-01764-s001.zip › Supplementary.pdf]

Supplementary Table S1. Primers used in this study.

Gene	Primer name	Primer sequences	Tm (°C)
REEP1	REEP1-F	ACAATTCGTGGTGACAGCAGCAG	60
	REEP1-R	GGAGTCGGCATACTGAGCAACAC	
ST6GAL1	ST6GAL1-F	CAGCCTAACCCACCGTCTTCAG	60
	ST6GAL1-R	GTCCGTCTGCCGCTTAGAAGG	
ASB15	ASB15-F	AGCTGGCTATGTGGGAAACGTC	60
	ASB15-R	GGAGTGGAGTCTCGCCTTTGTC	
EIF5B	EIF5B-F	GATGATGACCGCACCAAGGAAGAG	60
	EIF5B-R	GGGCACATTAGTCGCACCTATCTG	
IQSEC3	IQSEC3-F	GCAGGACAAGGAGAAGGAAGAAGG	60
	IQSEC3-R	TGGACACCGAGGTAGACGATGAG	
SUOX	SUOX-F	TGGAAGGTGGCGAGGCTGATG	60
	SUOX-R	GTCGGGCTGCACGTTGTAACTG	
NEURL1	NEURL1-F	TCTGGGCTTTGATTGATGTCTACGG	60
	NEURL1-R	CGCAGGCAGTCAGGAGGAATG	
RBM15	RBM15-F	AGGGAGCGACTGGTGGGAAAG	60
	RBM15-R	CTGCGGAGCGGTTGTCTGATG	
β-actin	β-actin-F	CAGCCATCTTTCTTGGGTAT	60
	β-actin-R	CTGTGATCTCCTTCTGCATCC	

Supplementary Table S2. The list of 189 DEGs.

gene_name	gene_chr	gene_length	gene_biotype
FRMD3	Z	3419	protein_coding
ENSGALG00000048328	1	550	lncRNA
ENSGALG00000050667	AADN05001176.1	1225	lncRNA
SUOX	33	1377	protein_coding
ENSGALG00000053624	25	917	lncRNA
MRPL4	30	1807	protein_coding
RBM15	26	2868	protein_coding
ENSGALG00000048802	2	294	protein_coding
ASB15	1	1854	protein_coding

-	10	531	-
NEURL1	6	3537	protein_coding
CCS	33	1182	protein_coding
ENSGALG00000055106	33	2405	lncRNA
ENSGALG00000054496	31	1889	protein_coding
SAPCD2	17	1566	protein_coding
ENSGALG00000046412	27	2289	protein_coding
ENSGALG00000048310	7	1320	protein_coding
-	KZ626833.1	425	-
MED11	KZ626839.1	354	protein_coding
ENSGALG00000054402	KZ626839.1	2450	protein_coding
STX1A	19	2590	protein_coding
HMGA1	26	378	protein_coding
EIF5B	1	4437	protein_coding
ENSGALG00000049557	33	3997	protein_coding
FIS1	15	1686	protein_coding
ENSGALG00000037112	2	2612	protein_coding
-	3	536	-
ENSGALG00000053749	6	309	protein_coding
ENSGALG00000053285	25	2025	protein_coding
-	KZ626833.1	449	-
ENSGALG00000054453	2	3212	lncRNA
SAMD5	3	468	protein_coding
ATP6V0D2	2	1760	protein_coding
ENSGALG00000050691	2	195	protein_coding
IQGAP2	Z	5846	protein_coding
-	22	2397	-
ENSGALG00000042375	2	8587	protein_coding
EVA1B	23	1173	protein_coding
ENSGALG00000049369	5	2338	protein_coding
ENSGALG00000046	33	8129	protein_coding

210			
ALPL	21	1603	protein_coding
TXLNB	3	4840	protein_coding
-	33	1807	-
MYL6	33	1603	protein_coding
ENSGALG00000054667	16	2133	protein_coding
ENSGALG00000047565	33	2247	protein_coding
ENSGALG00000054310	18	3246	protein_coding
ENSGALG00000053046	7	1515	protein_coding
HAX1	25	1426	protein_coding
ENSGALG00000031572	33	2317	protein_coding
ENSGALG00000029783	AADN05001439.1	1344	protein_coding
ENSGALG00000054941	1	1222	lncRNA
ALDH1L2	1	3492	protein_coding
ENSGALG00000052702	9	288	protein_coding
PTGS2	8	5101	protein_coding
ENSGALG00000048475	KZ626835.1	3552	pseudogene
FOXC1	2	1572	pseudogene
ENSGALG00000053925	AADN05001161.1	4012	protein_coding
REEP1	4	3261	protein_coding
ENSGALG00000050575	11	750	pseudogene
ENSGALG00000048280	2	1644	protein_coding
NNF1	23	2287	protein_coding
RBM8A	25	492	protein_coding
OPCML	24	1624	protein_coding
DHRS7C	18	1174	protein_coding
ENSGALG00000041560	KZ626826.1	5650	protein_coding
ENSGALG00000047953	KZ626837.1	768	protein_coding
ENSGALG00000049992	4	1335	lncRNA

ENSGALG00000051993	2	1965	protein_coding
ENSGALG00000026449	12	381	protein_coding
PDLIM4	13	1331	protein_coding
TINAGL1	23	1528	protein_coding
VAV3	8	5150	protein_coding
SLA	2	2971	protein_coding
-	1	1048	-
FLNA	27	4840	protein_coding
JCHAIN	4	1815	protein_coding
ADAM19	13	2703	protein_coding
ENSGALG00000015822	1	2100	protein_coding
LSM1	22	756	protein_coding
ENSGALG00000042151	4	1969	protein_coding
ENSGALG00000048899	30	288	protein_coding
-	12	9964	-
ENSGALG00000022531	5	465	protein_coding
ZFPM2	2	3564	protein_coding
ENSGALG00000051922	5	11985	pseudogene
-	32	602	-
CITED2	3	902	protein_coding
ENSGALG00000041565	5	291	protein_coding
ENSGALG00000050166	14	6355	protein_coding
ENSGALG00000048773	33	2439	protein_coding
CDC26	17	1255	protein_coding
GOLGA4	2	7646	protein_coding
ID4	2	1134	protein_coding
ENSGALG00000014857	Z	2378	protein_coding
OGN	12	2135	protein_coding
MMP13	1	2845	protein_coding
GDF11	33	1678	protein_coding
MYOM2	3	5286	protein_coding
ENSGALG00000031262	3	3524	protein_coding

EMILIN3	20	2664	protein_coding
ANKRD1	6	3202	protein_coding
ENSGALG00000049232	33	1513	protein_coding
ENSGALG00000048077	10	588	protein_coding
SLC4A10	7	3606	protein_coding
ALX4	5	1164	protein_coding
MYL10	19	1415	protein_coding
ENSGALG00000045898	7	3303	protein_coding
-	33	1153	-
LRRC39	8	1588	protein_coding
KBTBD11	3	2336	protein_coding
COL8A2	23	2439	protein_coding
C3AR1	1	1873	protein_coding
gga-mir-145	13	85	miRNA
5_8S_rRNA	16	153	rRNA
-	1	1168	-
GDF7	3	1203	protein_coding
ENSGALG00000005814	Z	2037	protein_coding
CCR2	2	3650	protein_coding
CCDC141	7	5308	protein_coding
-	30	1517	-
LVRN	Z	4092	protein_coding
ENSGALG00000054366	31	3347	protein_coding
IL18	24	2112	protein_coding
ENSGALG00000047981	32	1968	protein_coding
ENSGALG00000047805	30	1266	protein_coding
ENSGALG00000050395	19	1839	protein_coding
OTULIN	2	1084	protein_coding
ENSGALG00000047027	2	2915	protein_coding
ENSGALG00000048991	KZ626826.1	3203	protein_coding
TLR15	3	3341	protein_coding
SYP	31	1881	protein_coding
HDDC3	10	598	protein_coding
-	5	3081	-

-	AADN05001253.1	315	-
LMOD3	12	1575	protein_coding
ENSGALG00000015203	28	1664	protein_coding
TAF4B	2	2597	protein_coding
ENSGALG00000051467	25	5451	lncRNA
APCDD1L	20	1491	protein_coding
IQSEC3	1	3749	protein_coding
-	2	602	-
ENSGALG00000031447	22	1014	pseudogene
PLN	3	159	protein_coding
ENSGALG00000011177	1	2238	protein_coding
ENSGALG00000052818	11	1869	protein_coding
CDK20	13	2311	protein_coding
ENSGALG00000054572	12	1119	protein_coding
-	33	1028	-
ENSGALG00000007123	4	2112	protein_coding
TRPM2	9	5508	protein_coding
MYOT	13	2307	protein_coding
ABCG2	6	3026	protein_coding
SOX18	20	1585	protein_coding
SMPX	1	3083	protein_coding
ENSGALG00000050494	KZ626819.1	2864	protein_coding
VPS9D1	11	1506	protein_coding
PPFIBP2	5	4697	protein_coding
POU3F4	4	744	protein_coding
ENSGALG00000047526	5	1059	protein_coding
ENSGALG00000035685	MT	76	Mt_tRNA
TSPAN31	33	1029	protein_coding
-	15	1448	-
ENSGALG00000040995	7	7842	protein_coding
REPS2	1	5572	protein_coding
GNAZ	15	2821	protein_coding

MYOZ1		6	837	protein_coding
ENSGALG00000050325		26	4427	lncRNA
ENSGALG00000019934		3	393	protein_coding
SNORD127		5	86	snoRNA
ENSGALG00000054086		14	525	protein_coding
ENSGALG00000002511		26	1845	protein_coding
ENSGALG00000026553	Z		13242	protein_coding
FKBP2		33	1500	protein_coding
TMEM184A		14	1760	protein_coding
PPP1R3A		1	4163	protein_coding
KCND2		1	1899	protein_coding
ENSGALG00000048343		11	2636	protein_coding
ST6GAL1		9	3926	protein_coding
-		2	1285	-
NRCAM		1	7836	protein_coding
ENSGALG00000046160		4	1166	protein_coding
ENSGALG00000047158		7	1613	lncRNA
ENSGALG00000054048		19	1028	protein_coding
NTM		24	2818	protein_coding
ASCL3		5	1997	protein_coding
HPSE		4	2383	protein_coding
TDRKH		25	1680	protein_coding
ENSGALG00000047993		3	295	protein_coding
